# Intracellular Isotope Localization in *Ammonia* sp. (Foraminifera) of Oxygen-Depleted Environments: Results of Nitrate and Sulfate Labeling Experiments

**DOI:** 10.3389/fmicb.2016.00163

**Published:** 2016-02-19

**Authors:** Hidetaka Nomaki, Joan M. Bernhard, Akizumi Ishida, Masashi Tsuchiya, Katsuyuki Uematsu, Akihiro Tame, Tomo Kitahashi, Naoto Takahata, Yuji Sano, Takashi Toyofuku

**Affiliations:** ^1^Department of Biogeochemistry, Japan Agency for Marine-Earth Science and TechnologyYokosuka, Japan; ^2^Geology and Geophysics Department, Woods Hole Oceanographic InstitutionWoods Hole, MA, USA; ^3^Department of Chemical Oceanography, Atmosphere and Ocean Research Institute, The University of TokyoKashiwa, Japan; ^4^Department of Marine Biodiversity, Japan Agency for Marine-Earth Science and TechnologyYokosuka, Japan; ^5^Marine WorksYokosuka, Japan; ^6^Project Team for Research and Development of Next-generation Technology for Ocean Resources Exploration, Japan Agency for Marine-Earth Science and TechnologyYokosuka, Japan

**Keywords:** foraminifer, nitrate, NanoSIMS, electron dense body, endobionts, ultrastructure, denitrification

## Abstract

Some benthic foraminiferal species are reportedly capable of nitrate storage and denitrification, however, little is known about nitrate incorporation and subsequent utilization of nitrate within their cell. In this study, we investigated where and how much ^15^N or ^34^S were assimilated into foraminiferal cells or possible endobionts after incubation with isotopically labeled nitrate and sulfate in dysoxic or anoxic conditions. After 2 weeks of incubation, foraminiferal specimens were fixed and prepared for Transmission Electron Microscopy (TEM) and correlative nanometer-scale secondary ion mass spectrometry (NanoSIMS) analyses. TEM observations revealed that there were characteristic ultrastructural features typically near the cell periphery in the youngest two or three chambers of the foraminifera exposed to anoxic conditions. These structures, which are electron dense and ~200–500 nm in diameter and co-occurred with possible endobionts, were labeled with ^15^N originated from ^15^N-labeled nitrate under anoxia and were labeled with both ^15^N and ^34^S under dysoxia. The labeling with ^15^N was more apparent in specimens from the dysoxic incubation, suggesting higher foraminiferal activity or increased availability of the label during exposure to oxygen depletion than to anoxia. Our results suggest that the electron dense bodies in *Ammonia* sp. play a significant role in nitrate incorporation and/or subsequent nitrogen assimilation during exposure to dysoxic to anoxic conditions.

## Introduction

Marine sediments play important roles in biogeochemical cycles because the organic matter derived from the water column are mostly degraded or mineralized at the sediment-water interface prior to burial. During degradation, various chemical, and biological processes occur within the overlying oxic water and in anoxic layers of the sediments. Foraminifera are dominant components in terms of both abundance and biomass in benthic marine ecosystems in dysoxic to anoxic sediments (e.g., Bernhard et al., 2000; Gooday et al., 2000), and play a major role in the degradation of organic matter (Woulds et al., 2007). Because oxygen-depleted environments in the ocean have recently been expanding as a result of global warming (Shaffer et al., 2009; Keeling et al., 2010; Stramma et al., 2010), knowledge of benthic foraminiferal ecology in dysoxic to anoxic habitats is crucial for understanding relevant biogeochemical cycles on the seafloor.

Some benthic foraminifera have the ability to denitrify (Risgaard-Petersen et al., 2006; Høgslund et al., 2008; Bernhard et al., 2012a). Many benthic foraminiferal species are now known to respire nitrate, or at least to possess an intracellular nitrate pool (Piña-Ochoa et al., 2010; Nomaki et al., 2015). Foraminiferal denitrification has been estimated to be responsible for 4–70% of the total benthic denitrification (reviewed by Koho and Piña-Ochoa, 2012), although the estimate has high uncertainty due to methodological limitations (Kamp et al., 2015). Thus, knowledge of foraminiferal nitrate storage and related metabolisms, such as incorporation and localization of nitrate storage, rates of denitrification using an intracellular nitrate pool, and possible assimilation of remaining nitrate into biomass, is crucial for understanding benthic nitrogen cycles.

Recently, Nomaki et al. (2014) reported that the shallow-water benthic foraminifer *Ammonia* sp. incorporates nitrate in the seawater and the nitrogen isotopic composition (δ^15^N) of their amino acids reflects both isotopic compositions of nitrate and subsequent nitrate consumption in their cell. When isotopically labeled (either slightly ^14^N-enriched or ^15^N- enriched) nitrate was added to the overlying water of sediments containing *Ammonia* sp., the δ^15^N values of amino acids extracted from the whole foraminiferal cell showed a strong relationship with the δ^15^N values of the added nitrate, indicating an incorporation of nitrate into the foraminifera. Furthermore, the δ^15^N of amino acids were enriched in ^15^N by ~50%0, probably reflecting a preferential utilization of lighter nitrate (^14^NO${}_{3}^{-}$) from the intracellular nitrate pool, and the remaining nitrate pool becomes enriched in ^15^N as shown for other foraminiferal species by Bernhard et al. (2012a). Nomaki et al. (2014) speculated that the preferential utilization of lighter nitrate is attributed to denitrification either by host foraminifera or endobionts, because such ^15^N-enrichments were not found in specimens from the oxic condition (Nomaki et al., 2014). The nitrogen isotopic composition of amino acids in foraminiferal cells further suggested a synthesis of amino acids using an intracellular nitrate pool. These findings suggested that the foraminiferal intracellular nitrate pool is not only for denitrification by foraminifera (Risgaard-Petersen et al., 2006) or associated microbes (Bernhard et al., 2012b), but for assimilation of inorganic nitrogen into foraminiferal or bacterial biomass. The amino acid synthesis using the intracellular nitrate pool is likely performed by endobionts in foraminiferal cells, because the δ^15^N values of amino acids extracted from the foraminiferal test protein were almost constant regardless of label addition (Nomaki et al., 2014). However, those isotope measurements were carried out at the whole-cell level, so the localization of amino acid synthesis and the role of prokaryotic associates of foraminifera remain unclear.

Foraminiferal symbioses with endobionts or ectobionts under oxygen-depleted conditions have widely been reported from shallow water to deep-sea environments (e.g., Richardson and Rützler, 1999; Bernhard et al., 2000, 2010a, 2012b; Bernhard, 2003; Tsuchiya et al., 2015). Possible roles of those microbes have been discussed based on their phylogenetic relationships, functional genes of host foraminifera and endobionts, ultrastructural observations, and/or environmental conditions of their habitat. For instance, *Virgulinella fragilis*, which typically inhabits micro-oxic environments, retain δ-proteobacteria together with kleptoplasts (Tsuchiya et al., 2015). The phylogenetic analysis indicated that the endobiont had a similarity to the sulfate-reducing δ-proteobacteria, suggesting *V. fragilis* adapts to micro-oxic environments together with sulfate reducers. Thus, it is expected that sulfur is transferred from surrounding seawater to the host foraminifer or prokaryotic associates, in addition to nitrogen assimilation originated from nitrate. However, there is no report regarding elemental transfers between endobionts and foraminifera, due to the difficulties of elemental or isotopic measurements at spatial resolutions that discriminate endobionts vs. the host foraminifer.

Nanometer-scale secondary ion mass spectrometry (NanoSIMS) is a powerful tool for mapping elemental compositions and isotopic ratios at sub-micrometer resolution. NanoSIMS analysis has been applied to a wide variety of sample types, including carbonates (e.g., Sano et al., 2005; Shirai et al., 2008; Sano et al., 2012; Hori et al., 2015), meteorites and rocks (e.g., Besmehn and Hoppe, 2003; Mostefaoui and Hoppe, 2004), and organisms (e.g., Lechene et al., 2007; Popa et al., 2007; Morono et al., 2011). Recently, correlative NanoSIMS-TEM analyses have been performed to compare isotopic compositions and ultrastructure directly on semi-thin sections prepared for TEM (Finzi-Hart et al., 2009; Carpenter et al., 2013; Kopp et al., 2015). This correlative method enables us to examine assimilation of N and S originated from labeled nitrate or sulfate into organelles or endobionts, by comparing isotopic compositions and TEM images from ~100-nm-thick sections directly. In this study, we performed isotopic composition measurements using NanoSIMS on semi-thin sections of *Ammonia* sp., which had been incubated with ^15^N-nitrate and ^34^S-sulfate in either dysoxia or anoxia, after TEM observation on the same semi-thin sections to identify foraminiferal organelles and endobionts. We hypothesized that the endobionts in *Ammonia* sp. would be labeled with ^15^N as they seemed to synthesize amino acids using the foraminifer's intracellular nitrate pool (Nomaki et al., 2014). Further, if *Ammonia* sp. endobionts are allied with *V. fragilis'* endobionts, there may be elemental sulfur or other sulfur compounds originated from ^34^S-sulfate that can be confirmed with NanoSIMS. We present ultrastructural features of the *Ammonia* sp. incubated in either anoxia or dysoxia, present data regarding assimilation of ^15^N and ^34^S originated from added nitrate and sulfate, and discuss potential roles of possible endobionts and some organelles in nitrogen and sulfur metabolisms in dysoxic to anoxic conditions.

## Materials and methods

### Sediment sample collection

Surface sediments including *Ammonia* sp. were collected from the Nojima tidal flat (~0–1 m water depth) of Tokyo Bay on March 21, 2014. At the time of sampling in early spring, the sediment surface was heterogeneously covered by a brownish diatom mat. *Ammonia* sp. was mainly distributed in the top 1 cm of the sediment, within and beneath the diatom mat. Approximately the top 1 cm of sediment was scooped into several 4 L buckets together with ambient seawater. In the laboratory, the sediment samples were kept in the buckets with a sediment thickness of 4–5 cm; they were illuminated to allow diatom growth at the sediment surface. Visible gastropods and crustaceans were removed from the buckets to improve the water conditions and avoid predation on foraminifera.

### Isotope labeling experiment

We prepared both dysoxic and anoxic incubation conditions. Surface sediment samples containing benthic foraminifera were sieved through a 1-mm mesh. The finer fractions were mixed and split among three 140-mL clear glass bottles to a depth of ~4 cm and three 50-mL syringes to a depth of ~ 5 cm. The tip of the 50-mL syringes was removed and the plunger's silicone plug was placed in the cut-off end to seal the core bottom. The bottles and syringes were then filled with seawater that had been passed through a 0.22 μm filter. The three glass bottles were tightly closed without headspace gas to prevent oxygenation of overlying water during the experiment; hereafter we call them the “anoxic” condition. The syringes were placed in a rack without any cap to allow oxygen diffusion through the air–water interface to create dysoxic conditions in the surface sediments. All of the bottles and syringes were placed in a sealed plastic box to avoid evaporation and were maintained at 20°C, which is a suitable temperature for their growth. The anoxic condition bottles were gently shaken 10 times a day to homogenize the sediments and environment, while the syringes were kept stable. The overlying water of the anoxic bottles became anoxic 2–3 days after incubation initiation, as confirmed by an oxygen microelectrode (Unisense, Denmark).

Among the three bottles or syringes in each treatment, one of them was kept as a control without stable isotope addition during the experiment. To the overlying water in each of the other four bottles or syringes, we added 0.8 mL of filtered seawater (salinity = 32) containing Na^15^NO_3_ (^15^N atom% = 99%) to a final concentration of 50 μmol L^−1^, and Na_2_^34^SO_4_ (^34^S atom% = 98%) to a final concentration of 4 mmol L^−1^, after first removing the same volume of seawater. Those labeled compounds were added every 3 or 4 days, and incubations were terminated after 14 days.

The incubation period, 14 days, was selected because the nitrate-originating N assimilation into foraminiferal amino acids were detected after 1–2-month incubations of similar experiments (Nomaki et al., 2014). Another *in situ* experiment confirmed that deep-sea foraminifera synthesize fatty acid and sterols using ^13^C derived from ^13^C-labeled algae within 4 or 6 days, but not within 2 days (Nomaki et al., 2009). Although the lipid synthesis and amino acid synthesis involve different pathways, and the ^13^C-labeled algae had to be catabolized before it became available (thus required more time than nitrate), these observations suggested that foraminifera require several days to synthesize organic matter at a detectable level with isotope ratio mass spectrometry (IRMS). Because NanoSIMS requires much more labeling of ^15^N or ^34^S compared to IRMS, we chose a 14-day incubation to ensure that foraminifera incorporate nitrate and synthesize substantial amounts of ^15^N or ^34^S compounds for the NanoSIMS measurements.

At the end of the experiment, dissolved oxygen concentrations were measured from ~5 mm above the sediment surface to 10-mm depth in the sediment in 200-μm steps with microelectrodes with a tip diameter of 100 μm using an automatic profiler controlled by Sensor Trace Pro software (Unisense, Denmark) on a Windows PC. Overlying water was then gently removed by 20-mL syringe, filtered with a 0.45-μm filter, and kept frozen at −30°C in 50-mL plastic tubes for the determination of nutrient concentration and ^34^S concentration in sulfate. For the dysoxic incubations, sediment depths corresponding to 0.1–22 μmol L^−1^ O_2_ based on microelectrode profiles were sampled by pushing up the sediment core from the bottom of the syringe; hereafter we define these as the “dysoxic” condition. Sediments were fixed in 10% glutaraldehyde in cacodylate buffer to a final concentration of 4.0% glutaraldehyde. Specimens whose test, except for the last one or two chambers, were filled with bright yellow cytoplasm typical of this species were selected for TEM observation. About 10 specimens per condition were fixed with 3.0% glutaraldehyde in 0.1 M cacodylate buffer overnight at 20°C and stored at 4°C prior to further processing.

The same fixation and preparation of foraminifera were also carried out using natural sediment samples to examine the isotopic compositions of non-manipulated specimens.

### Cellular ultrastructure observations and EDS analyses

Sample fixation, staining with uranium, and subsequent embedding followed the protocol for TEM sample preparation at JAMSTEC (Nomaki et al., 2014, 2015; Tsuchiya et al., 2015). In brief, about 20 specimens of fixed foraminifera were embedded in 1% aqueous agarose and then cut into ~1-mm cubes. Samples were decalcified with 0.2% EGTA in 0.81 mol L^−1^ aqueous sucrose solution (pH 7.0) for several days, rinsed with filtered seawater, and then postfixed with 2% osmium tetroxide in filtered seawater for 2 h at 4°C. Samples were rinsed with an 8% aqueous sucrose solution and stained *en bloc* with 1% aqueous uranyl acetate for 2 h at room temperature. Stained samples were rinsed with distilled water, dehydrated in a graded ethanol series, and embedded in Quetol 651 resin (Nisshin EM, Tokyo, Japan).

All foraminiferal specimens were sectioned into semi-thin section (500-nm thick) using an ultramicrotome (Ultracut S, Leica) and observed using a light microscope (BX51, Olympus) to confirm the cytoplasmic condition. Three or four specimens among 10 embedded specimens from anoxic and dysoxic conditions, respectively, were further sectioned into ultra-thin sections (60-nm thick) and semi-thin sections (200-nm thick). The sections were stained with 2% aqueous uranyl acetate and lead stain solution (0.3% lead nitrate and 0.3% lead acetate, Sigma-Aldrich), and were observed by TEM (Tecnai 20, FEI) at an acceleration voltage of 120 kV. Ultra-thin sections were used for detailed observations of ultrastructure, e.g., organelle membranes and cristae of mitochondria. After confirming the specimen appeared to have intact organelles (i.e., had been live at the time of fixation), the semi-thin sections of two specimens from both anoxic and dysoxic conditions were further observed with TEM. The periphery of the cell and surrounding parts in the newest two or three chambers were observed sequentially and composite TEM images were reconstructed into a mosaic on a computer using Adobe Photoshop CS5.

Also, we performed energy-dispersive X-ray spectroscopy (EDS) to quantitatively analyze relative compositions of S in different organelles. A 200-nm-semi-thin section was cut from a single specimen of *Ammonia* sp. incubated under anoxia. A semi-thin section not subject to TEM was coated with a 20-nm thick layer of carbon. Elemental compositions of electron dense bodies, the organic lining, possible endobionts, and mitochondria were measured using the EDAX EDS system (EDAX Inc.) supplied with the TEM.

### NanoSIMS analyses and isotopic ratio calculations

The semi-thin sections (200-nm thick) of four specimens (two specimens from anoxia, and two specimens from dysoxia) previously imaged with TEM were coated with osmium to a thickness of 20 nm to avoid electron charging during the NanoSIMS analysis. The semi-thin sections were set into a sample folder of a nanometer-scale secondary ion mass spectrometer (NanoSIMS NS50; AMETEK, Inc., CAMECA SAS), installed at the Atmosphere and Ocean Research Institute, the University of Tokyo. We performed two types of measurements for each area of each semi-thin section: ^15^N/^14^N analysis and ^34^S/^32^S analysis. For ^15^N/^14^N analysis, a ~1 pA primary beam of Cs^+^ was focused on a < 600 nm diameter spot. Secondary ions were extracted by an accelerating voltage of 8 kV, and the ^12^C^−^, ^13^C^−^, ^12^C^14^N^−^, ^12^C^15^N^−^, and ^32^S^−^ ions were measured simultaneously using a multi-collector system. The peak of ^12^C^15^N^−^ was separated from ^12^C^14^NH^−^ and ^13^C^14^N^−^ with adequate mass resolution of 3000 at 10% peak height. The peak of ^32^S^−^ was clearly separated from ^16^O_2_ in the same mass resolution. For ^34^S/^32^S analysis, a ~5 pA primary beam < 1-μm in diameter was used and ^13^C^−^, ^12^C^15^N^−^, ^32^S^−^, and ^34^S^−^ ions were measured simultaneously from the same area as the ^15^N/^14^N analysis. Typically, a 10 × 10-μm area of the semi-thin section was examined for each set of measurements with a 256 × 256 pixel raster; a total of 50 images were amalgamated for the data quantification. The total examined area was 1450 and 1100 μm^2^ for the two anoxic-incubated specimens and 801 and 225 μm^2^ for the two dysoxic-incubated specimens. In cases where the semi-thin section was damaged during the NanoSIMS measurements and the obtained image showed deformation of the semi-thin section, we removed that data from subsequent data analyses. During the ^34^S analysis, sometimes a 128 × 128 pixel raster and 25 images at each area were performed to reduce potential damage to the semi-thin sections.

We quantified isotopic compositions of the following organelles and ultrastructural features: vacuoles, food vacuoles, mitochondria, peroxisomes, plastids, lipid droplets, possible endobionts, electron dense bodies, cytosome with no obvious organelles, and organic lining as well as the surrounding resin. For the calculation of the isotopic ratios, we compared counts of mass number 26 (^12^C^14^N^−^) with composite TEM images, since the mass number 26 compared well with the foraminiferal ultrastructure. A 256 × 256-pixel grid was aligned to each organelle or ultrastructural feature; averaged values of ^15^N and ^34^S atom% were calculated from ^12^C^15^N^−^/(^12^C^14^N^−^+^12^C^15^N^−^) and ^34^S^−^/(^32^S^−^+^34^S^−^) data from corresponding pixels, respectively. During this quantification, we confirmed that there was no relationship between total count numbers of ^15^N or ^34^S and variations in isotopic compositions. The ^15^N atom% values were also measured in natural foraminiferal samples that were similarly processed; these data were compared with the ^15^N atom% measured with an isotope ratio mass spectrometer coupled with an elemental analyzer (EA/IRMS). The averaged nitrogen isotopic compositions of natural samples measured with NanoSIMS and bulk nitrogen isotopic compositions measured with EA/IRMS were 0.3751 atom% (δ^15^N of 24.0%0to AIR) and 0.3714 atom% (δ^15^N of 10.1%0to AIR), respectively. We did not perform a comparison of sulfur isotopic compositions between NanoSIMS and conventional methods. The ^34^S atom% measured in this study, therefore, cannot be considered as absolute values due to matrix effects and other factors (Hoppe et al., 2013), but can be used for determining relative labeling patterns between dysoxic vs. anoxic conditions, and between different ultrastructure and organelles.

To test the significance of differences in isotopic compositions of each organelle or ultrastructural feature, a Mann–Whitney test was used comparing those in anoxic vs. dysoxic conditions. A Kruskal–Wallis test was applied to test the differences in isotopic compositions among organelle and ultrastructural features. When this test detected a significant difference among these, a Steel–Dwass test was conducted to identify which combination showed the significant differences. These statistical tests were performed with R, version 3.1.3 (R Development Core Team, 2008), using the package *coin* (Hothorn et al., 2015). Code of the Steel–Dwass test was obtained from the website (http://www.trifields.jp/introducing-steel-dwass-in-r-1632).

We note that the isotopic compositions measured in this study, using NanoSIMS on semi-thin sections, only reflect the insoluble phase of the foraminiferal cytoplasm. The soluble phase likely was lost during processing and embedding into resin. Therefore, nitrate and sulfate directly stored as foraminiferal vacuole contents, and soluble compounds synthesized through nitrate and sulfate, such as free amino acids, were not evaluated with the present method.

## Results

### Ultrastructural characteristics

**Figure 1:**
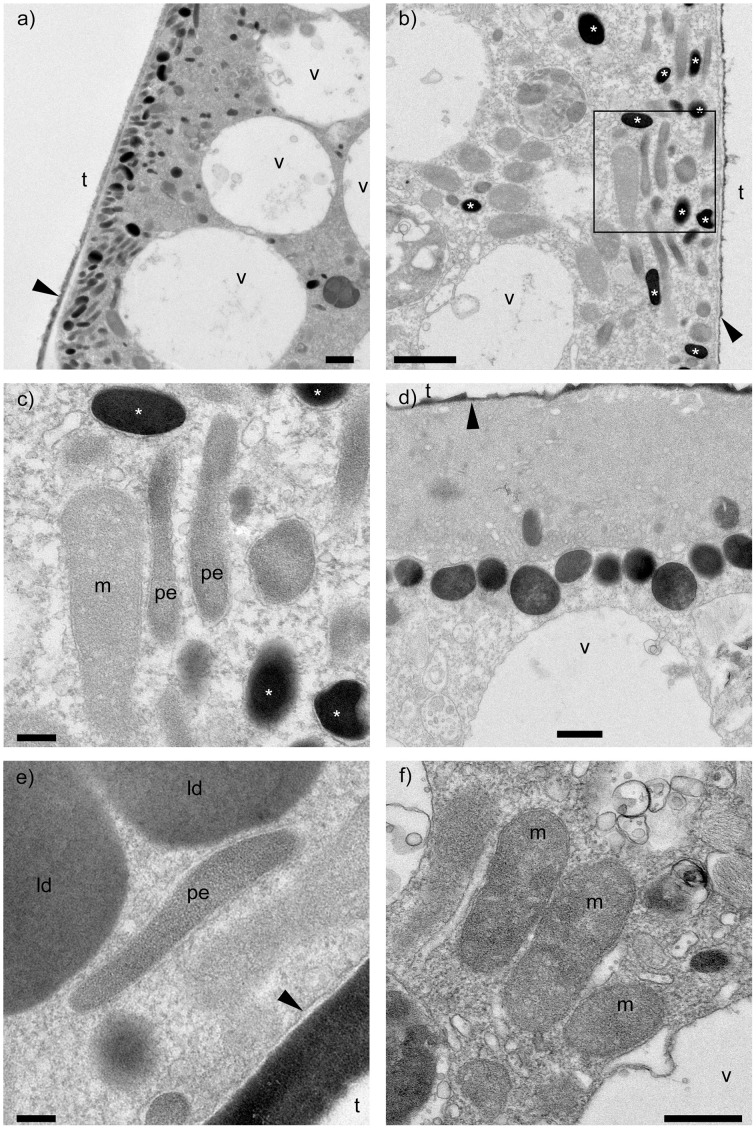
**Transmission electron micrographs of *Ammonia* sp. incubated with anoxic conditions**. **(A,B)** Clusters of electron dense bodies (^*^) found near the cell periphery of the antepenultimate and penultimate chambers, respectively. **(C)** Close-up view of outlined area in b showing electron dense bodies (^*^), most bound by membrane. **(D)** Electron dense bodies showing heterogeneous electron densities. **(E)** Close-up view of a possible endobiont surrounded by membrane. **(F)** Mitochondria showing double exterior membrane. m, mitochondria; pe, possible endobionts; v, vacuoles; t, test (former location); ld, lipid droplet; black arrows, organic lining. Scale bars: **(A,B)** = 1 μm; **(C,E)** = 200 nm; **(D,F)** = 500 nm. Panels **(A–C,F)**: anoxic specimen A; panels **(D,E)**: anoxic specimen B.

Specimens incubated in anoxia showed a consistent and prominent arrangement of ultrastructure in their youngest (newest, most recently formed) two or three chambers of all three observed specimens. Electron dense bodies, which typically appeared black in TEM images, were concentrated at the cell periphery (Figures 1A,B). They were ~200–500 nm in diameter, spherical to spheroidal, and some of them appeared to be membrane bound (Figure 1C). The electron density occasionally varied inside these electron dense bodies, sometimes exhibiting granule-like structures (Figure 1D). These electron dense bodies were also found in the dysoxic-incubated specimens, however, they were not clustered at the cell periphery, appearing scattered through the cytoplasm of the four observed specimens.

Interestingly, putative endobionts similar to those observed by Nomaki et al. (2014) in other *Ammonia* sp. specimens incubated in a similar manner were not observed in our study. Instead, possible endobionts, which were elongated with diameters of ~0.1–0.3 μm and lengths sometimes >1 μm (Figure 1E), co-existed with electron dense bodies, commonly near the cell periphery (Figures 1B,C). Some of these structures appeared to be membrane bound, although these membranes were not as apparent as those around other organelles such as mitochondria (Figure 1F). Also, the interiors of our possible endobionts did not show typical bacterial structures like ribosomes or nucleoids. Similar ultrastructural features (i.e., electron dense bodies, possible endobionts) were also observed in specimens incubated in dysoxia but without clustering at the cell periphery.

### Elemental and isotopic mapping using NanoSIMS

**Figure 2:**
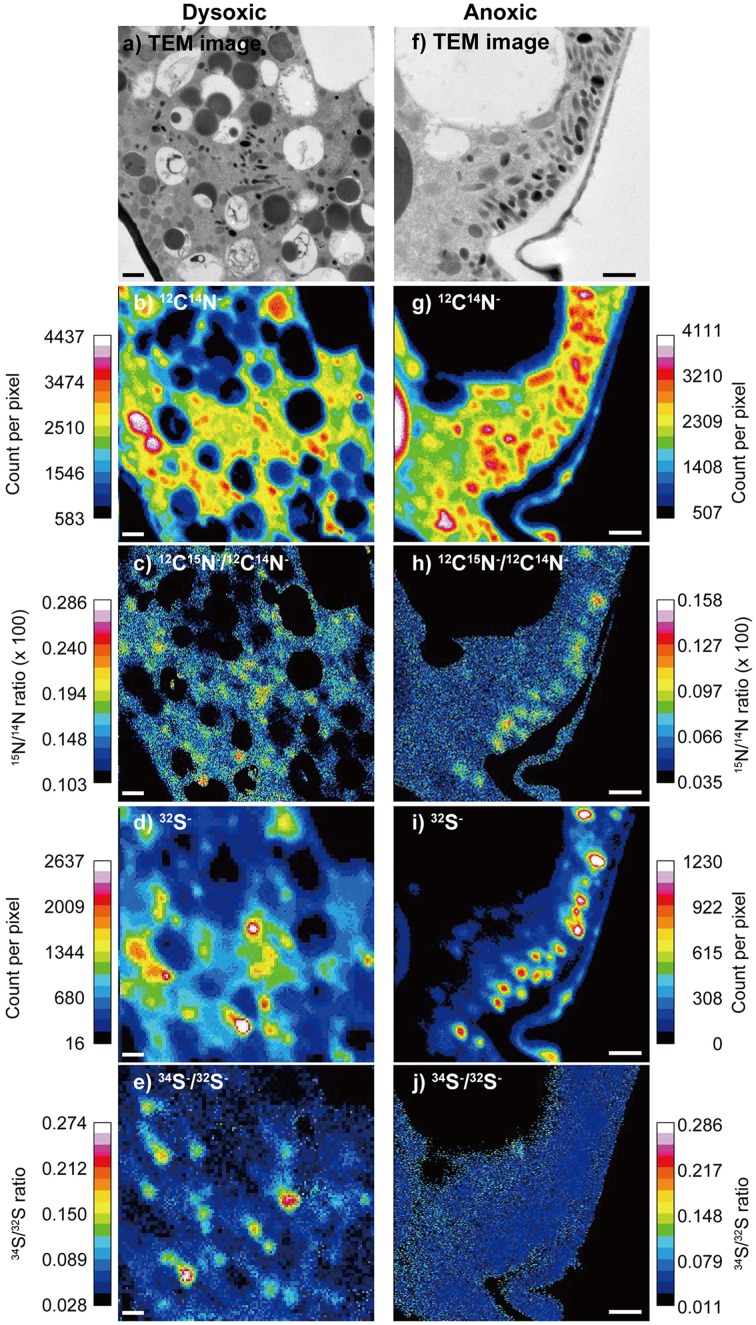
**An example of a TEM image and corresponding NanoSIMS images from a dysoxic-incubated (left column) and anoxic incubated (right column) *Ammonia* sp. specimen**. **(A,F)** TEM image of the measured area (12 × 12 μm for **A** and 8 × 8 μm for **F**), **(B,G)** ^12^C^14^N^−^ counts, **(C,H)** ^12^C^15^N^−∕12^C^14^N^−^ ratio, **(D,I)** ^32^S^−^ counts, and **(E,J)** ^34^S^−^/^32^S^−^ ratio. During the ratio processing of **(C,E,H,J)**, pixels where ^12^C^14^N^−^ counts below 1000 or pixels where ^32^S^−^ counts below 60 were removed to reduce noises. Note that the images **(D,E)** were obtained with 128 × 128 pixel raster, while others were obtained with 256 × 256 pixel raster. Scales = 1 μm.

The distributional patterns of ^12^C^14^N^−^ counts corresponded well to electron densities appearing in TEM images (Figure 2). In the surrounding resin and vacuoles, the ^12^C^14^N^−^ concentrations were low because the resin polymer solution contains trace amounts of nitrogen. Lipid droplets also exhibited low ^12^C^14^N^−^ counts, in comparison to other organelles and the cytosome. The distribution of ^32^S^−^ counts presented distinct concentrations at most of the electron dense bodies.

**Figure 3:**
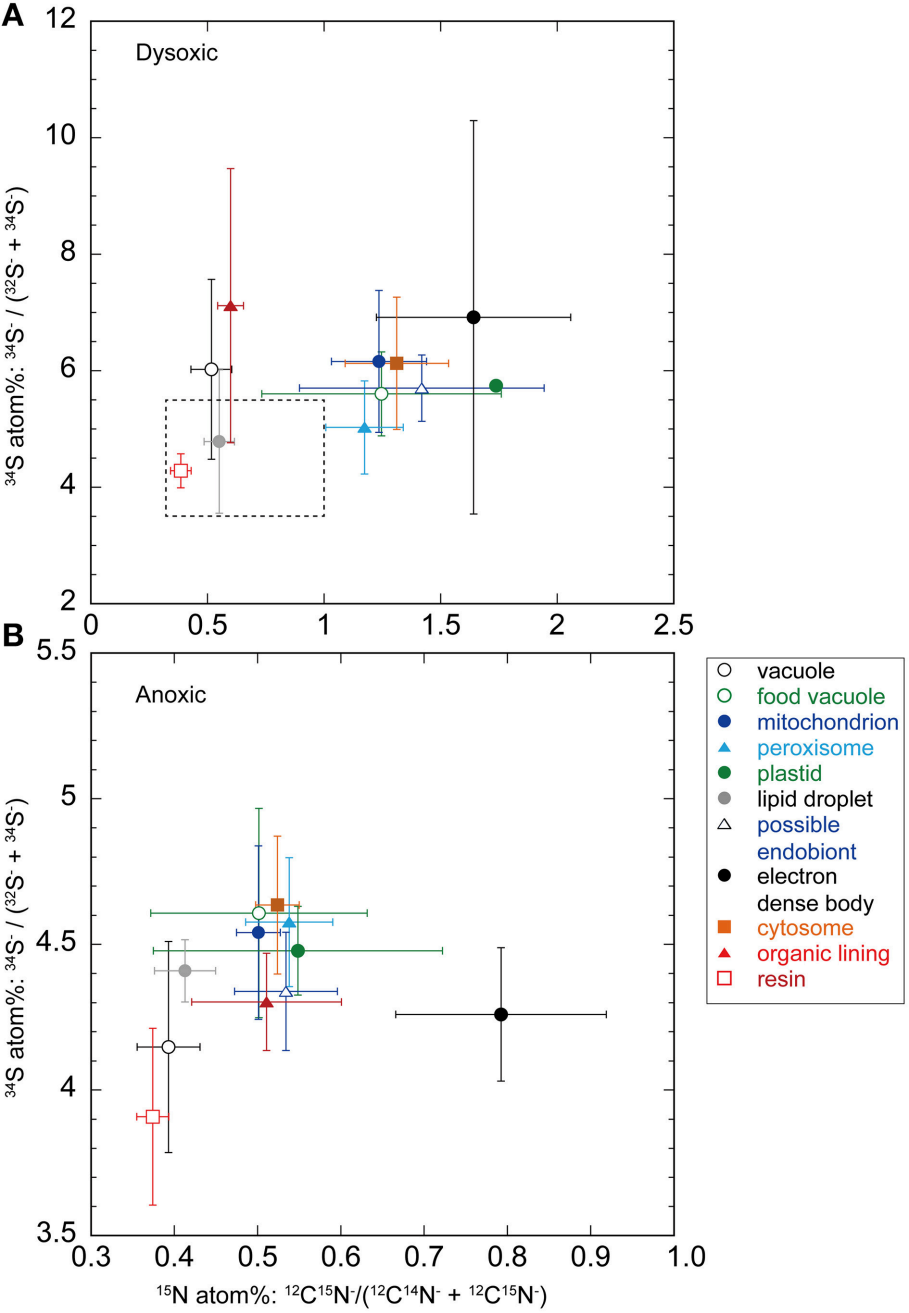
**Averaged (± SD) nitrogen and sulfur isotopic compositions (in atomic %) of organelles and ultrastructural features from foraminiferal semi-thin sections (A, dysoxic specimens; B, anoxic specimens)**. Note different scales on both sets of axes. The dotted box in a corresponds to the same scale that shown in **(B)**.

Some ultrastructural features were clearly labeled with both ^15^N and ^34^S in our 2-week dysoxia-incubated specimens (Figures 2C,E). In our anoxic-incubated specimens, labeling with ^15^N was also observed (Figure 2H) while ^34^S-labeling was not apparent (Figure 2J). Results obtained from all four examined specimens indicated that the extents of ^15^N and ^34^S labeling of each organelle and ultrastructure were significantly higher in dysoxic specimens than in anoxic specimens, with some exceptions: no significant differences in ^15^N for resin, no significant differences in peroxisome and lipid droplets for ^34^S between dysoxic and anoxic (Figure 3, Tables 1, 2). The extents of ^15^N and ^34^S labeling among organelles and ultrastructural features significantly differed in each condition (Kruskal–Wallis tests, ^15^N in dysoxia: χ^2^ = 125.07, *p* < 0.001; ^15^N in anoxia: χ^2^ = 185.53, *p* < 0.001; ^34^S in dysoxia: χ^2^ = 45.22, *p* < 0.001; ^34^S in anoxia: χ^2^ = 97.15, *p* < 0.001). Steel–Dwass tests detected the significant differences in ^15^N or ^34^S labeling between different organelles or ultrastructural features among each condition (Supplementary Tables 1–4).

**Table 1:** **Results of Mann–Whitney test of ^15^N atom% between dysoxic and anoxic conditions**.

	***n***		^**15**^**N atom%**		**Mann–Whitney test**	
	**Dysoxic**	**Anoxic**	**Dysoxic**	**Anoxic**	**Z**	***p***
Vacuole	27	31	0.52 ± 0.09	0.39 ± 0.04	−5.87	< 0.001
Mitochondrion	25	52	1.24 ± 0.20	0.5 ± 0.03	−7.07	< 0.001
Organic lining	9	15	0.60 ± 0.06	0.51 ± 0.09	−2.06	< 0.05
Electron dense body	21	44	1.64 ± 0.42	0.79 ± 0.13	−5.44	< 0.001
Food vacuole	11	17	1.25 ± 0.51	0.5 ± 0.13	−4.12	< 0.001
Possible endobiont	9	28	1.42 ± 0.52	0.53 ± 0.06	−4.46	< 0.001
Cytosome	11	36	1.31 ± 0.22	0.52 ± 0.03	−4.98	< 0.001
Peroxisome	5	4	1.17 ± 0.17	0.54 ± 0.05	−2.45	< 0.05
Lipid droplet	27	9	0.55 ± 0.07	0.41 ± 0.05	−4.18	< 0.001
Resin	12	15	0.39 ± 0.04	0.37 ± 0.02	−1.00	>0.05

**Table 2:** **Results of Mann–Whitney test of ^34^S atom% between dysoxic and anoxic conditions**.

	***n***		^**34**^**S atom%**		**Mann–Whitney test**	
	**Dysoxic**	**Anoxic**	**Dysoxic**	**Anoxic**	**Z**	***p***
Vacuole	27	31	6.03 ± 1.54	4.15 ± 0.36	−5.53	< 0.001
Mitochondrion	25	52	6.16 ± 1.22	4.54 ± 0.30	−6.47	< 0.001
Organic lining	9	15	7.12 ± 2.35	4.3 ± 0.17	−4.02	< 0.001
Electron dense body	21	44	6.92 ± 3.38	4.26 ± 0.23	−5.23	< 0.001
Food vacuole	11	17	5.60 ± 0.72	4.61 ± 0.36	−3.32	< 0.001
Possible endobiont	9	28	5.70 ± 0.57	4.34 ± 0.20	−4.46	< 0.001
Cytosome	11	36	6.13 ± 1.14	4.63 ± 0.24	−4.87	< 0.001
Peroxisome	5	4	5.03 ± 0.80	4.58 ± 0.22	−1.47	>0.05
Lipid droplet	27	9	4.79 ± 1.23	4.41 ± 0.22	0.09	>0.05
Resin	12	15	4.28 ± 0.29	3.91 ± 0.30	−2.73	< 0.01

Theoretically, the resin should not be labeled with either ^34^S or ^15^N because specimens were embedded in resin after the isotope labeling experiment. This was true in our specimen preparations to some extent both for ^15^N (0.39 ± 0.05 atom% and 0.37 ± 0.02 atom% in dysoxia and anoxia, respectively) and ^34^S (4.28 ± 0.29 atom% and 3.94 ± 0.29 atom% in dysoxia and anoxia, respectively; Figure 3), except there was slight enrichment in ^15^N in dysoxia. Significant differences were found in ^34^S atom% of resin between dysoxic and anoxic (Mann–Whiteney test, *p* < 0.01, Table 2) with some variation in ^34^S atom% (Supplementary Figure 1). Thus, ^34^S labeling of the other structures should be interpreted with some degree of caution.

The ^15^N and ^34^S atom% of vacuoles of anoxic specimens were not significantly different from those of resin in anoxic incubation (Steel–Dwass test, *p* > 0.05, see Supplementary Tables 3, 4): 0.39 ± 0.04 ^15^N atom% and 4.15 ± 0.36 ^34^S atom%, respectively. Vacuoles of dysoxic specimens were slightly enriched in ^15^N (0.52 ± 0.09 atom %) and apparently enriched in ^34^S (6.03 ± 1.54 atom %) compared to their resin, and these differences were significant (Steel–Dwass test, *p* < 0.05, Supplementary Tables 1, 2). In both dysoxic and anoxic conditions, both the cytosome, where no obvious organelles existed, and mitochondria exhibited similar labeling patterns. These features were significantly labeled with more ^15^N and ^34^S under dysoxia (1.31 ± 0.22 ^15^N atom% and 6.13 ± 1.14 ^34^S atom% for cytosome; 1.24 ± 0.20 ^15^N atom% and 6.16 ± 1.22 ^34^S atom% for mitochondria) than under anoxia (0.52 ± 0.03 ^15^N atom% and 4.63 ± 0.24 ^34^S atom% for cytosome; 0.50 ± 0.03 ^15^N atom% and 4.54 ± 0.30 ^34^S atom% for mitochondria; Mann–Whitney test, *p* < 0.001 for both ^15^N and ^34^S of cytosome and mitochondria between dysoxic and anoxic, Tables 1, 2). Although few peroxisomes were analyzed, their isotopic compositions were similar to those of mitochondria and the cytosome in both conditions (Figure 3) and no significant difference was detected between mitochondria and cytosome in both ^15^N and ^34^S atom% under both anoxia and dysoxia (Supplementary Tables 1–4).

The highest enrichments in ^15^N were observed in the electron dense bodies in specimens from both dysoxic (1.64 ± 0.42 atom%) and anoxic conditions (0.79 ± 0.13 atom%). Electron dense bodies were significantly labeled more with ^15^N in comparison to other examined organelles and ultrastructure in anoxia, except plastid and peroxisome which the numbers of the measurements were low (Supplementary Table 3). Electron dense bodies were only slightly labeled with ^34^S under anoxia (4.26 ± 0.23 atom%) in comparison to resin, but did label with ^34^S after the dysoxia incubation (6.92 ± 3.38 atom%, Supplementary Tables 2, 4). However, there was a large variation of the ^34^S enrichments under dysoxia; some electron dense bodies were labeled up to 15 atom%, and some were not enriched above resin levels (Supplementary Figure 1A). Large variations were also observed in ^15^N labeling of electron dense bodies, both in dysoxia and in anoxia (Figure 3, Supplementary Figure 1).

In both dysoxic and anoxic conditions, the possible endobionts were labeled with ^15^N with large variations, and some of them exhibited higher ^15^N atom% than in the cytosome or mitochondria (Supplementary Figure 1), although the averaged values were not statistically different (Supplementary Tables 1, 3). Conversely, ^34^S atom% of possible endobionts under dysoxia were significantly lower than those of the cytosome or mitochondria (Figure 3A, Supplementary Table 4), suggesting the possible endobionts have a different metabolism than the holobiont at least in terms of sulfur.

The organic lining was slightly enriched in ^15^N in both conditions: 0.60 ± 0.06 atom% and 0.51 ± 0.09 atom% under dysoxia and anoxia, respectively. Apparent enrichments in ^34^S were recorded only under dysoxia, where values ranged from 5.15 to 12.4 atom%. All measured organic linings were labeled with ^34^S in dysoxia, suggesting synthesis of the organic lining during the 2-week incubation using S derived from the added sulfate, while some electron dense bodies were not labeled with ^34^S (Supplementary Figure 1).

### Differences between chambers

We performed NanoSIMS analysis on the youngest three chambers of specimens incubated in anoxia, where the electron dense bodies and possible endobionts were observed. For a given specimen, among those youngest three chambers, there was no obvious variation in either the ^15^N and ^34^S enrichments (Supplementary Figure 2). The electron dense bodies, which were typically labeled with ^15^N in anoxic conditions, were labeled with ^15^N in similar ranges (0.4–1.1 ^15^N atom%) among the last, penultimate and antepenultimate chambers of a specimen incubated under anoxia (Supplementary Figure 2). The ^34^S atom% of the electron dense bodies also exhibited similar ranges between chambers, although the number of measurements on electron dense bodies in the last chamber was small.

### S contents measured with EDS

Among organelles and ultrastructural features examined with EDS, the electron dense bodies contained the highest relative S content, which was 0.34 ± 0.25% among the sum of C, O, and S, on average (Table 3). The organic lining also contained substantial amounts of S (0.13 ± 0.14%), but with large spatial variations. Possible endobionts and mitochondria exhibited low amounts of S (0.02 ± 0.02% and 0.00 ± 0.01%, respectively) in comparison to the electron dense bodies and organic linings.

**Table 3:** **Relative abundances (atomic %) of C, O, and S in *Ammonia* sp. incubated with anoxia**.

**Element**	**Electron dense body (*n* = 4)**	**Organic lining (*n* = 4)**	**Possible endobiont (*n* = 4)**	**Mitochondrion Mitochondrion (*n* = 7)**
C	98.4 ± 0.87	98.5 ± 1.60	98.7 ± 0.05	99.0 ± 090
O	1.24 ± 0.65	1.39 ± 1.47	1.30 ± 0.06	0.99 ± 0.90
S	0.34 ± 0.25	0.13 ± 0.14	0.02 ± 0.02	0.00 ± 0.01

Note that both C and O were also derived from the resin, while the S was derived only from original foraminiferal cytoplasm.

## Discussion

### Ultrastructural differences compared to previous experiments

The ultrastructure observed in this study exhibited notable differences from that observed in previous experiments using same foraminiferal species and same incubation conditions (Nomaki et al., 2014). In the previous study, rod-shaped endobionts, ~0.3–0.5 μm in diameter and 1.2–1.8 μm in length were observed typically near vacuoles, often between two or three vacuoles. In this study, thinner possible rod-shaped endobionts with diameters of ~0.1–0.3 μm were observed near the cell periphery in the youngest three chambers. Our possible endobionts co-existed with clusters of electron dense bodies near the cell periphery of all three observed anoxia specimens (Figure 1).

The major difference between Nomaki et al. (2014) and this study is the season of the sediment sampling. Sediment samples for the present isotope labeling experiment were collected in March, while the sediment samples for Nomaki et al. (2014) were collected in June and July. Through February and March 2014, the atmospheric temperature sometimes decreased below 2°C and was usually below 18°C in the daytime (Japan Meteorological Agency, http://www.data.jma.go.jp/gmd/risk/obsdl/index.php). This is far colder than the temperature ranges in June and July, which generally ranged between 15–25°C and 20–30°C, respectively. The large differences in temperature between March and June/July may result in different benthic microbial communities and their activities (e.g., Abdollahi and Nedwell, 1979; Hansen et al., 1981; Rooney-Varga et al., 1997), and also different metabolisms of *Ammonia* (Bradshaw, 1961). Considering the fact that there were no endobionts in specimens collected from natural sediments and in an oxic-incubated specimen in Nomaki et al. (2014), it is probable that *Ammonia* sp. of this area gain potential endobionts from the ambient sediments when conditions mandate. Thus, the variability of the microbial community in sediments may affect the adaptive ecology of *Ammonia* sp. The probable temporal symbiosis found in *Ammonia* sp. contrasts the consistent retention of δ-proteobacteria and kleptoplasts in *V. fragilis* (Bernhard, 2003; Tsuchiya et al., 2015). The inconsistent presence of endobionts may suggest that the symbiosis of *Ammonia* sp. is plastic, and able to accommodate variable conditions that they encounter while the symbiosis of *V. fragilis* is more robust. It is also possible that the symbiosis of *Ammonia* sp. was established recently, while the double symbiosis of *V. fragilis* has a relatively long history, being widespread among observed locales (i.e., Venezuela, Japan, New Zealand, Namibia).

Another possible explanation for the difference in endobionts is seasonal changes in physiological state. *Ammonia* sp. in the Nojima tidal flat typically reproduces asexually in March to April, and grows toward summer (Nomaki, unpublished data). This phenology suggests that the adult specimens in March are not in an active growth phase, but are nearing asexual reproduction. On the other hand, specimens in June to July may still require energy for their growth. Furthermore, the oxygen concentration in sediments decreases from March to July or August, due to higher temperatures and subsequent enhancement of microbial activities (Abdollahi and Nedwell, 1979). The seasonality in metabolic state of the populations and the changes in redox state may explain the variability in cell biology (i.e., endobiont presence/absence).

### Different labeling patterns in anoxic and dysoxic conditions

Labeling with ^15^N and ^34^S was more apparent in specimens incubated with dysoxia compared to the anoxia (Supplementary Figures 3, 4). This difference suggests either higher foraminiferal activity under dysoxia or increased availability of the ^15^N-nitrate and ^34^S-sulfate during exposure to dysoxia compared to anoxia. It is possible that the labeled substrates were transformed either biologically or chemically during our 2-week incubations. For instance, partial denitrification and dissimilatory reduction of nitrate to ammonium (DNRA) convert nitrate to nitrite and ammonium ion (Francis et al., 2007). Nitrogen cycling in marine sediments consists of a variety of compounds and pathways, which are mainly facilitated by prokaryotes (Libes, 2009). Among inorganic nitrogen, ammonium ion is a suitable inorganic nitrogen source for prokaryotic growth (Kirchman, 2012), resulting in production of microbial biomass or production of bacteriovore biomass which contains ^15^N. These processes that affect the availability of nitrate largely vary between presence or absence of oxygen (Canfield et al., 2010). When ^15^N-nitrate is added into anoxic conditions it can be reduced quickly (Nomaki et al., 2014). If the nitrate is used by denitrification or DNRA before foraminifera have an opportunity to use it, the ^15^N-nitrate in the experimental anoxic bottle could be less available compared to that available in dysoxic conditions. Such a change may explain the lower ^15^N-enrichments in our anoxic incubations compared to dysoxic incubations.

The concentration of seawater ^34^S-sulfate remained substantially unchanged in both dysoxic and anoxic conditions (33.5 and 33.6 atom% in the dysoxic and anoxic treatments, respectively). The differential enrichments in ^34^S of foraminifera between treatments were likely due to higher foraminiferal activities during exposure to dysoxic conditions compared to anoxic conditions.

Some foraminiferal species can survive in sediments below oxygen penetration depth or even during exposure to anoxic conditions (e.g., Bernhard, 1993; Moodley et al., 1997; Langlet et al., 2013, 2014). Nardelli et al. (2014) further reported growth (evaluated by chamber addition) of three calcareous foraminiferal species under anoxic conditions, and concluded that these foraminiferal species are not solely surviving under anoxia as dormant inhabitants. Our present incubation experiments also showed an incorporation and assimilation of N and S even under anoxic conditions into foraminiferal organelles (Figure 3), but these were less active in comparison to dysoxic conditions, where energy can be obtained through aerobic respiration. Further analysis of the metabolic activity of foraminifera under different redox states is required to understand their actual vitality during exposure to anoxic conditions.

### Possible role of electron dense bodies

The most apparent labeling with ^15^N was recorded in the electron dense bodies regardless of incubation condition (i.e., dysoxia or anoxia) (Supplementary Figure 5). This observation suggests that the electron dense bodies play an important role in foraminiferal nitrogen assimilation in oxygen-depleted environments. Although the electron dense bodies were slightly labeled with ^34^S in the anoxic specimens, electron dense bodies of dysoxic-incubated specimens were more enriched in ^34^S. As noted, the electron dense bodies have spherical-spheroidal shapes with 200–500 nm diameters and a surrounding membrane in some cases (Figures 1A–D). Our ^32^S^−^ mapping (Figures 2D,I) and EDS (Table 3) results show that electron dense bodies were rich in sulfur in comparison to other organelles or ultrastructural features, and that sulfur may be originated from seawater sulfate based on ^34^S labeling. The large variation in ^34^S-labeling among electron dense bodies in dysoxia may reflect the timing of their formation. No significant enrichment in ^34^S in anoxia relative to vacuoles (Figure 3, Supplementary Table 4, Supplementary Figure 6) suggests electron dense bodies were not formed using ^34^S-sulfate in anoxic incubations.

The electron dense bodies were commonly observed in *Ammonia* sp., not only in specimens exposed to anoxic conditions, but also in specimens exposed to dysoxia. Features similar to electron dense bodies appear in *Ammonia tepida* exposed to copper contamination (Le Carde and Debenay, 2006). Similar ultrastructural features were also observed in *Bulimina tenuata* from hydrocarbon seeps (Bernhard et al., 2010b), in *B. tenuata* from the dysoxic environment of Santa Barbara Basin (Bernhard and Bowser, 2008), and in *Planoglabratella opercularis* from rocky shores (Tsuchiya, unpublished data), although those size ranges varied and the presence/absence of a surrounding membrane was not documented. TEM sample preparation methods may account for differences in appearances (i.e., electron density) among these cases. We are confident that the high electron densities of *Ammonia* sp. electron dense bodies in our study reflect high contents of sulfur based on NanoSIMS and EDS analyses (Figure 2I, Table 3).

In anoxic specimens, the electron dense bodies were concentrated near the cell periphery of the youngest two or three chambers, often together with spheroidal to rod-shaped structures having diameters from 100 to 300 nm, which we interpret as putative endobionts, whereas electron dense bodies were scattered in dysoxic specimens. The concentrated distribution of electron dense bodies near the cell periphery of anoxia-incubated specimens suggests interaction with the environment and/or ambient seawater.

One potential explanation of the identity of the electron dense body is a kind of mitochondria-related organelle. For instance, a mitosome in *Entamoeba histolytica* has been reported to possess proteins involved in sulfate uptake and sulfate activation (Mi-ichi et al., 2009). In *E. histolytica*, the sulfate activation pathway is important for sulfur-containing lipid synthesis (Mi-ichi et al., 2011), which is crucial for *E. histolytica* survival, proliferation, and encystment (Mi-ichi et al., 2011, 2015). It is possible that the electron dense bodies in *Ammonia* sp. play a role similar to *E. histolytica* mitosomes, though *Ammonia* sp. possessed mitochondria (Figure 1F). Because *Ammonia* sp. electron dense bodies had high S concentrations and considerable labeling with ^34^S in dysoxia, it is possible that our *Ammonia* sp. also synthesized sulfolipids through a sulfate activation pathway. Such a pathway, which may facilitate encystment or dormancy of *Ammonia* sp., may explain the lower incorporation of S in anoxia-incubated specimens. Bernhard and Alve (1996) discussed the possibility of foraminiferal dormancy in response to surviving anoxia. They found that although the survival rates of some foraminifera (e.g., *Adercotryma glomeratum, Bulimina marginata*) were comparable between N_2_-incubated and aerated control treatments, the adenosine triphosphate (ATP) concentrations in the foraminiferans were significantly lower after anoxic exposure. This implies some foraminifera survive anoxia by reducing their metabolic activity as dormancy, while some other foraminiferal species survive oxygen depletion with denitrification (Risgaard-Petersen et al., 2006), symbiosis (Bernhard et al., 2000), and/or potential utilization of H_2_O_2_ (Bernhard and Bowser, 2008). Although their study was not subject to anoxia, 2 years of possible dormancy was also documented for shallow-water benthic foraminifera (Alve and Goldstein, 2010). Foraminiferal dormancy might be a common response to surviving unfavorable conditions such as seasonal anoxia, however, we need further dedicated studies on this topic.

It is also possible that the electron dense bodies are a type of peroxisome or microbody having a sulfate activation pathway, although the electron dense bodies are not similar in appearance to peroxisomes demonstrated to have catalase cores that were observed in foraminifera from naturally occurring oxygen-depleted habitats (Bernhard and Bowser, 2008). Further observations are required to better understand the identity of the electron dese body and its role(s) in foraminiferal adaptation to anoxia.

As noted, electron dense bodies were often at the cell periphery of the youngest three chambers along with possible endobionts that were labeled with ^15^N in some cases (Figures 1, 3). These enrichments observed in possible endobionts are concordant with the interpretation of the previous experiments to some extent, where Nomaki et al. (2014) speculated amino acid synthesis occurred in endobionts that used nitrate from the intracellular nitrate pool. However, the enrichments observed in this study, which are ~2 ^15^N atom%, were relatively low in comparison to the previous report where the nitrogen isotopic compositions of amino acids were almost comparable to that of added nitrate. Further, the sizes and distributions of endobionts differed between studies, suggesting presence of different microbe types. Finally, ^34^S enrichment was not significant in our possible endobionts, suggesting sulfur metabolism was not a major pathway in the endobionts observed in this study. Molecular phylogenetic analysis of endobionts are required to gain further insights about their identity and roles in holobiont metabolism.

### Methodological advantages and limitations

As shown in the results, correlative TEM and NanoSIMS analyses using semi-thin sections successfully indicated the elemental and isotopic compositions of foraminiferal organelles and ultrastructural features at submicron scales. This correlative approach has a wide range of potential applications regarding foraminiferal metabolism. For example, this methodology can assess the elemental transfer between algal symbionts, endobionts, and ectobionts, as well as the roles of some foraminiferal organelles such as kleptoplasts, peroxisomes, and lipid droplets.

In the present study, it is recognized that numbers of replicate specimens were low. This was because of the time consuming nature of the sample preparation, TEM observation, and isotopic composition measurements using NanoSIMS. More replicate measurements are required to enable confident generalization of our results. Furthermore, time series sampling using this correlative approach is required to better understand the actual pathways of label incorporation into foraminiferal cytoplasm. Our 14-day incubation period makes it difficult to precisely interpret whether the labeling with either ^15^N or ^34^S resulted directly from added nitrate or sulfate, or if the labeling resulted from other forms of nitrogen or sulfur compounds that were produced over the 2 weeks incubations.

The other limitation of the present correlative method is that soluble compounds in foraminiferal cells were likely lost during sample processing and embedding into resin, as mentioned in the Materials and Methods section. Therefore, nitrate and sulfate directly incorporated and stored in foraminiferal vacuole contents, or soluble compounds synthesized through nitrate and sulfate, such as free amino acids, were not evaluated with the present method. Application of cryofixation of foraminiferal specimens (Goldstein and Richardson, 2002) may overcome such solution change problem, and serve more direct elemental and isotopic mapping of the cell.

## Author contributions

HN, JB, MT, TT desingned this study. HN, JB, MT, TT, KU, and AT performed the TEM observations. HN, JB, AI, NT, and YS performed the NanoSIMS analysis. TK and HN performed the statistical analyses. All authors contributed to the correspondence data analyses and interpretations. All authors read and approved the final manuscript.

### Conflict of interest statement

The authors declare that the research was conducted in the absence of any commercial or financial relationships that could be construed as a potential conflict of interest.
