# Supplementary material for: Intracellular Isotope Localization in Ammonia sp. (Foraminifera) of Oxygen-Depleted Environments: Results of Nitrate and Sulfate Labeling Experiments
Source: Front Microbiol. 2016 Feb 19;7:163. doi: 10.3389/fmicb.2016.00163 (PMC4759270; doi:10.3389/fmicb.2016.00163)
Supplement: Supplementary file 5 [file DataSheet1.DOCX]

Supplementary Material

Intracellular isotope localization in Ammonia sp. (Foraminifera) of oxygen-depleted environments: results of nitrate and sulfate labeling experiments

**Hidetaka Nomaki*, Joan M. Bernhard, Akizumi Ishida, Masashi Tsuchiya, Katsuyuki Uematsu, Akihiro Tame, Tomo Kitahashi, Naoto Takahata, Yuji Sano, Takashi Toyofuku**

***Correspondence:** Hidetaka Nomaki: nomakih@jamstec.go.jp

## Supplementary Figures captions

Supplementary figure 1. Nitrogen and sulfur isotopic compositions (in atomic %) of organelles and ultrastructural features from foraminiferal semi-thin sections (a. dysoxic specimens; b. anoxic specimens). Note different scales on both sets of axes. The dotted box in a corresponds to that shown in b.

Supplementary figure 2. Nitrogen and sulfur isotopic compositions (in atomic %) of electron dense bodies measured from three different chambers of anoxic specimen A.

Supplementary figure 3. Differences in nitrogen isotopic compositions of each organelle and ultrastructural feature between dysoxic and anoxic incubated specimens.

Supplementary figure 4. Differences in sulfur isotopic compositions of each organelle and ultrastructural feature between dysoxic and anoxic incubated specimens.

Supplementary figure 5. Differences in nitrogen isotopic compositions among organelles and ultrastructural features from dysoxic-incubated specimens. The dotted line indicates the natural abundance of ^15^N.

Supplementary figure 6. Differences in sulfur isotopic compositions among organelles and ultrastructural features from dysoxic-incubated specimens. The dotted line indicates the natural abundance of ^34^S.

**
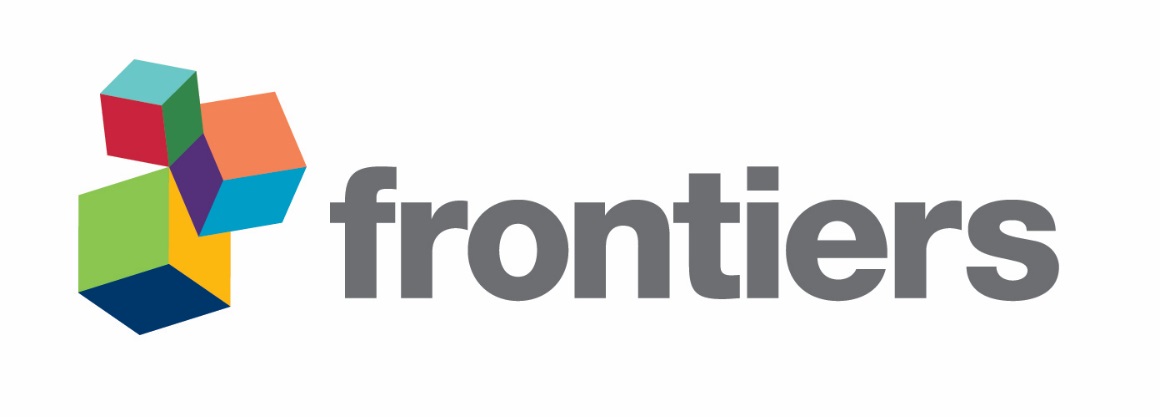
**

## Supplementary Tables

**Supplementary table 1.** Result of Steel-Dwass test of ^15^N atom% between each organelle and ultrastructure under dysoxic condition. Upper right and lower left indicate the t values and *p* values, respectively.

**Supplementary table 2.** Result of Steel-Dwass test of ^34^S atom% between each organelle and ultrastructure under dysoxic condition. Upper right and lower left indicate the t values and *p* values, respectively.

**Supplementary table 3.** Result of Steel-Dwass test of ^15^N atom% between each organelle and ultrastructure under anoxic condition. Upper right and lower left indicate the t values and *p* values, respectively.

**Supplementary table 4.** Result of Steel-Dwass test of ^34^S atom% between each organelle and ultrastructure under anoxic condition. Upper right and lower left indicate the t values and *p* values, respectively.
