# Supplementary figures and images for: Intracellular Isotope Localization in Ammonia sp. (Foraminifera) of Oxygen-Depleted Environments: Results of Nitrate and Sulfate Labeling Experiments
Source: Front Microbiol. 2016 Feb 19;7:163. doi: 10.3389/fmicb.2016.00163 (PMC4759270; doi:10.3389/fmicb.2016.00163)

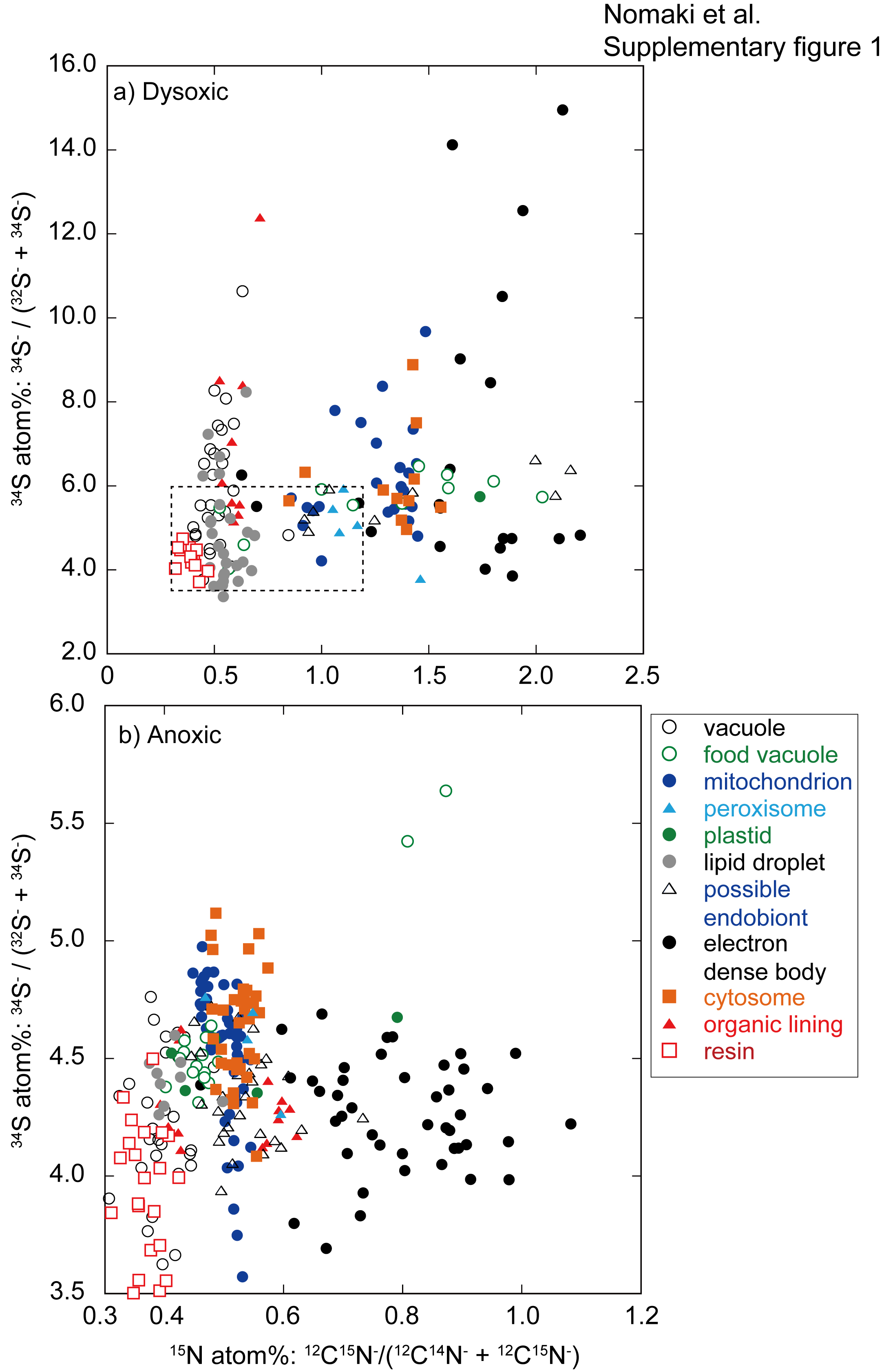

Supplement: Supplementary file 6 [file Image1.JPEG]

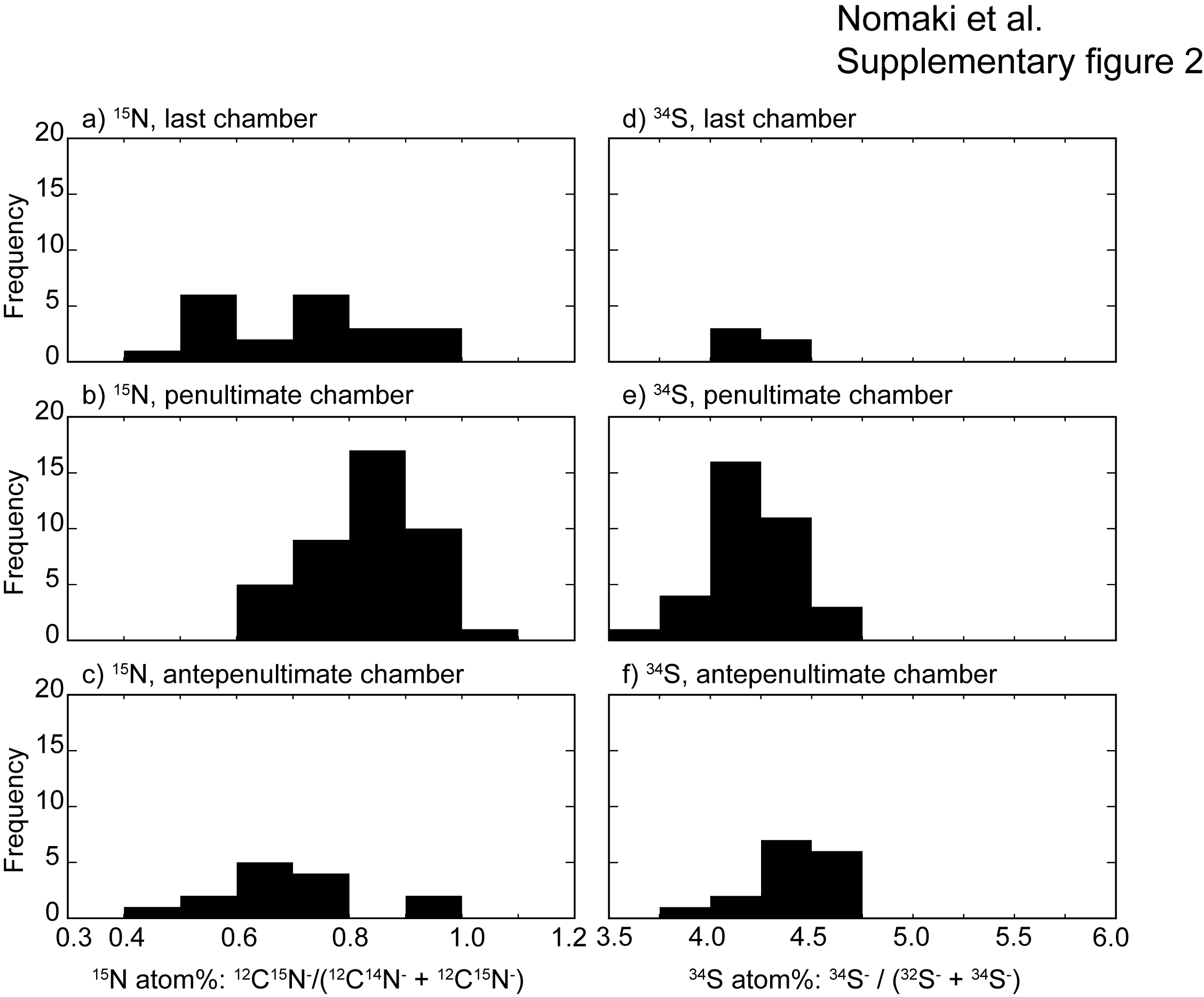

Supplement: Supplementary file 7 [file Image2.JPEG]

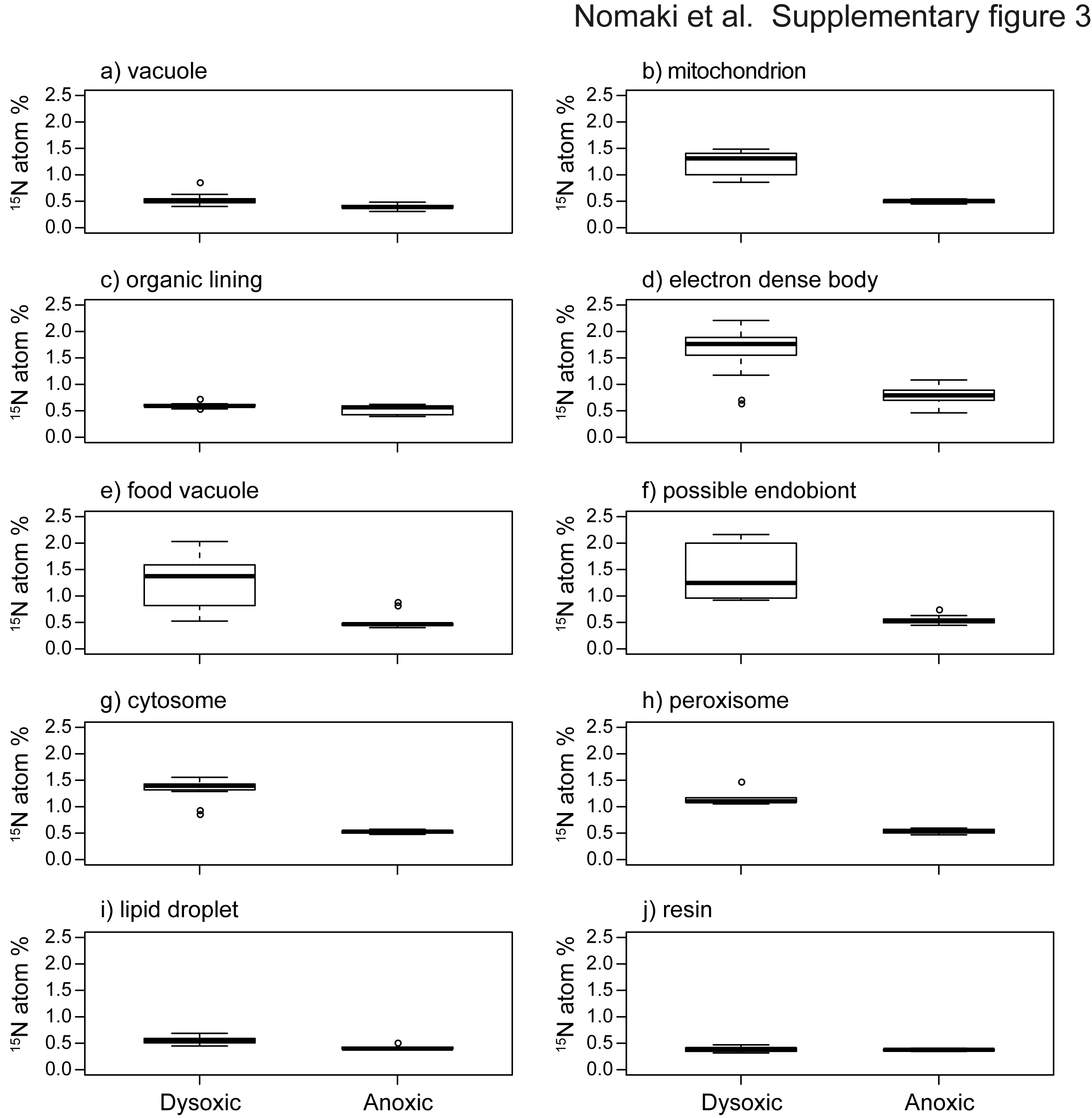

Supplement: Supplementary file 8 [file Image3.tif]

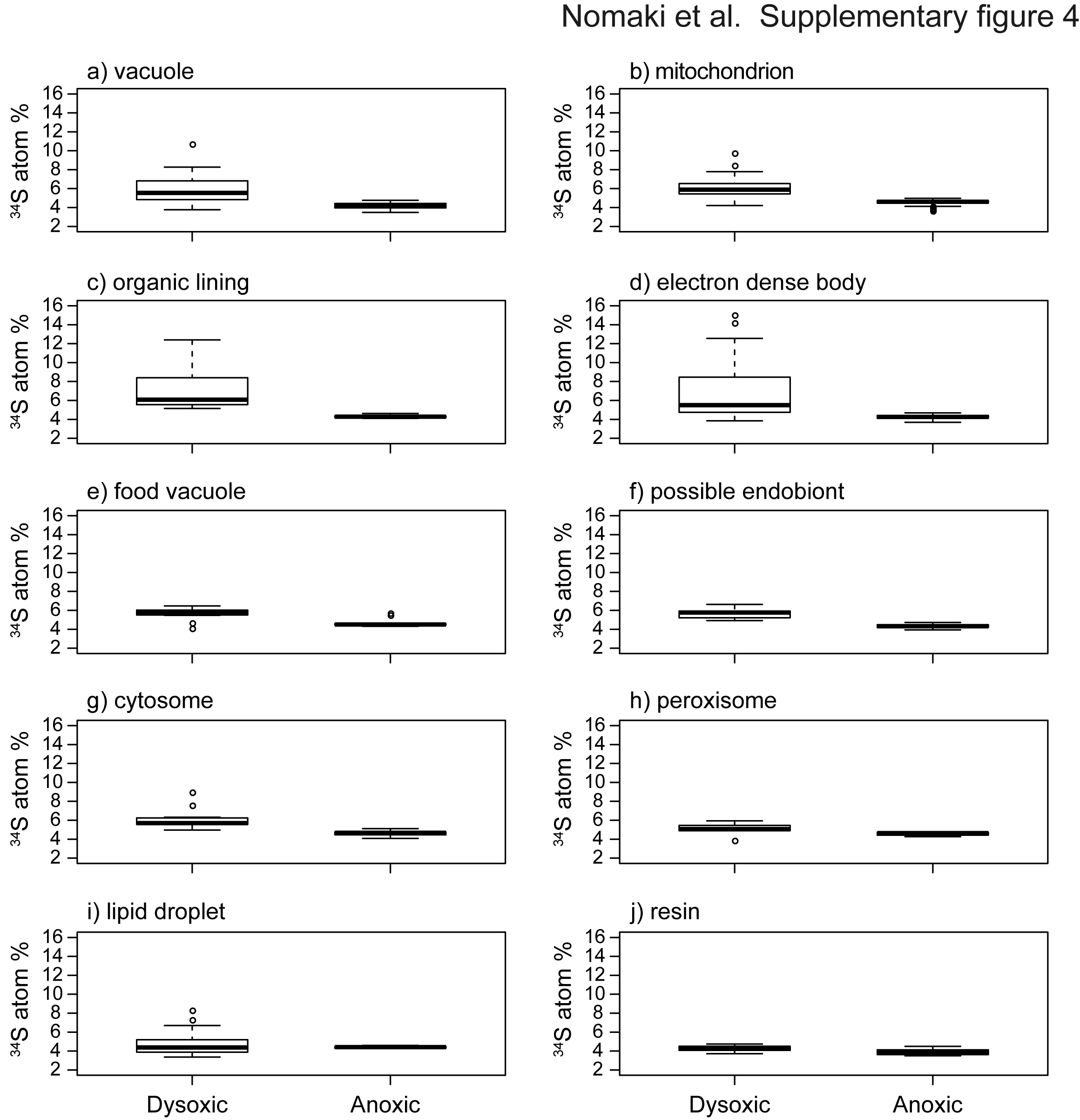

Supplement: Supplementary file 9 [file Image4.JPEG]

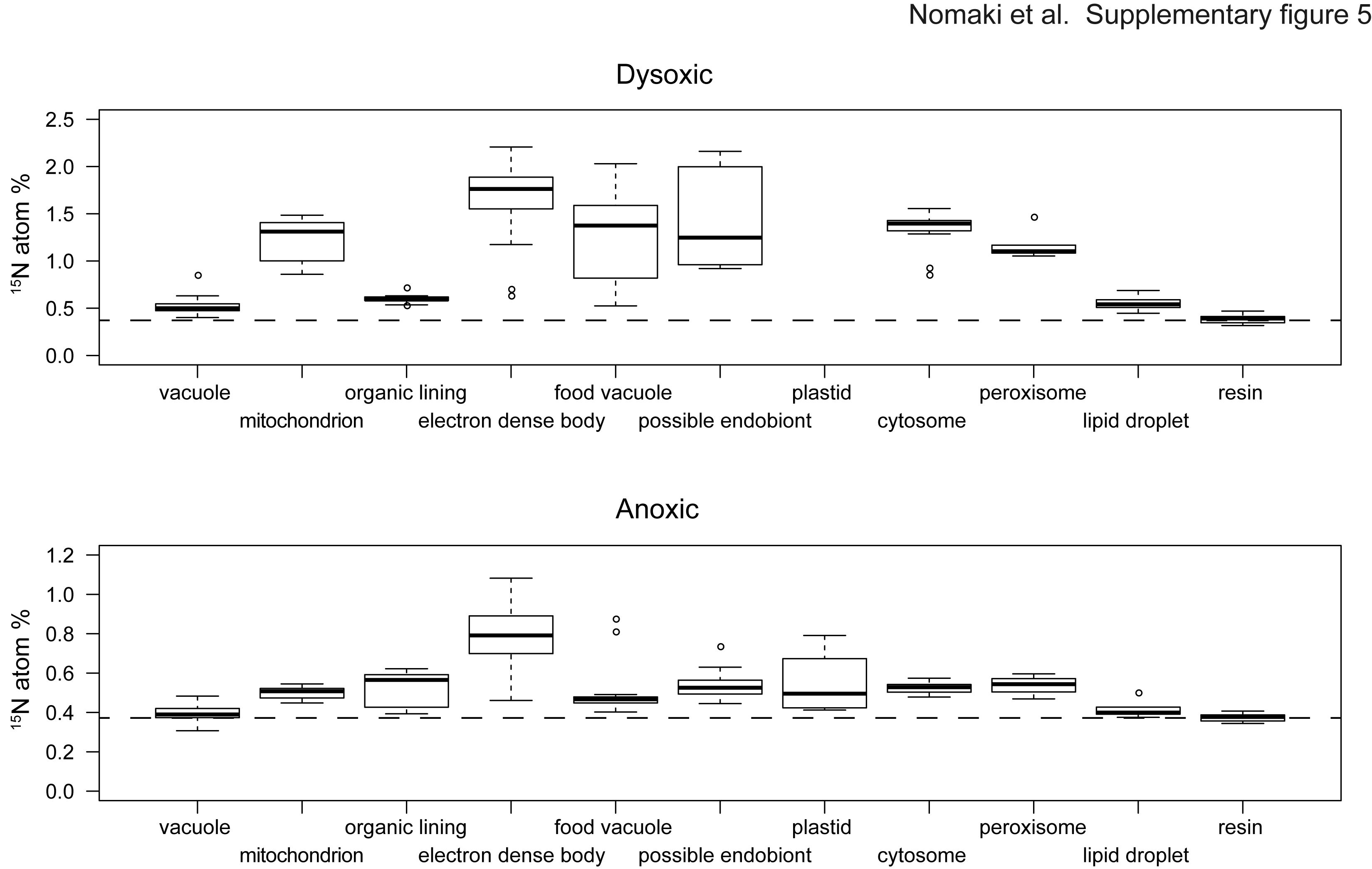

Supplement: Supplementary file 10 [file Image5.JPEG]

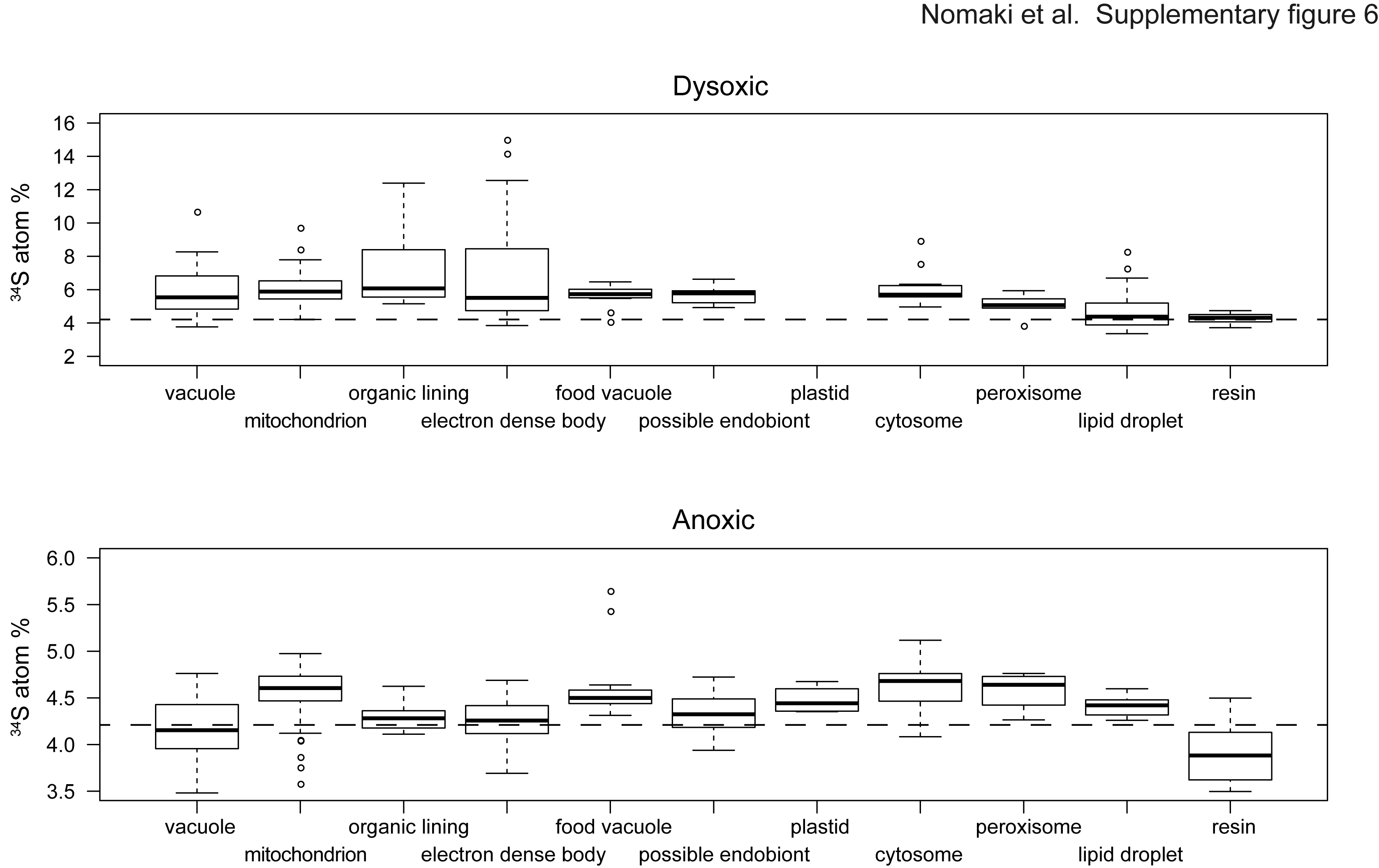

Supplement: Supplementary file 11 [file Image6.JPEG]
